# The association of agricultural and non-agricultural work on the healthy ageing of older adults in Japan: A 6-year longitudinal study from the Japan Gerontological evaluation study

**DOI:** 10.1016/j.pmedr.2024.102949

**Published:** 2024-12-13

**Authors:** Hiroki Takeuchi, Kazushige Ide, Hequn Wang, Motoki Tamura, Katsunori Kondo

**Affiliations:** a Advanced Preventive Medical Sciences, Graduate School of Medical and Pharmaceutical Sciences, Chiba University, Chiba-shi, Chiba, Japan; b Department of Social Preventive Medical Sciences, Center for Preventive Medical Sciences, Chiba University, Chiba-shi, Chiba, Japan; c Department of Community Building for Well-being, Center for Preventive Medical Sciences, Chiba University, Chiba-shi, Chiba, Japan; d Department of Community Health and Preventive Medicine, Hamamatsu University School of Medicine, Hamamatsu, Shizuoka, Japan

**Keywords:** Agricultural workers, Dementia, Healthy life expectancy, Age-friendly city, Age-friendly community, Sustainable development goals

## Abstract

Objectives: Many studies have examined the impact of employment on health, but few large-scale longitudinal studies specifically investigate the impact of agricultural labor on the health of older adults. This study aims to identify the health effects of employment on older Japanese adults, focusing on agricultural workers.

Methods: This study uses longitudinal data collected by the Japan Gerontological Evaluation Study (JAGES) from 2013 to 2019. We selected 48,221 older adults out of a total of 65,751 respondents, excluding missing values. The objective variables included healthy ageing, such as dementia, functional disability, loss of healthy life expectancy, and death. Explanatory variables were used to categorize participants into four groups: non-agricultural workers, agricultural workers, retired, and those who have never worked. Seven adjustment variables, including sex, age, and socioeconomic status, were considered. Logistic and modified Poisson regression analyses were employed after imputing missing values.

Results: Incidence ranged from 2.6 % (dementia) to 17.3 % (any level of functional disability). Post-multiple imputation analysis showed significantly lower odds ratios and risk ratios for dementia, functional disability, loss of healthy life expectancy, and death among non-agricultural and agricultural workers compared to retirees. The odds and risk ratios for agricultural workers ranged from 0.45 (dementia) to 0.69 (loss of healthy life expectancy).

Conclusions: Compared with retirees, non-agricultural and agricultural workers experience significantly reduced risks for dementia, functional disability, loss of healthy life expectancy, and death. These findings showed potential health benefits associated with continued employment in older age.

## Highlights

• This study examined the health effects of agricultural labor on older adults.
• Employment, including agriculture, was associated with better health outcomes compared to retirement.
• Agricultural workers showed a significantly reduced risk of mortality.
• Sustained agricultural work supports health, aligning with health goals of sustainable development goals.

## 1 Introduction

Improving older adults' health and well-being is a key goal of age-friendly city and community (AFCC) policies. Civic participation and employment have been identified as strategic interventions for healthy ageing (World Health Organization, 2018). These efforts align with the sustainable development goals (SDGs; United Nations, 2024) promoting the mental and emotional well-being of older adults by enabling them to continue working and achieve good health and well-being.

Previous studies have reported negative and positive health effects of agricultural work. Studies on agricultural workers have highlighted specific health risks, providing foundational data for clarifying the mixed health impacts of agricultural work. A study reported that ageing is associated with an increased risk of health issues among agricultural workers, such as injuries, musculoskeletal symptoms, and stress (Beseler and Rautiainen, 2023). Furthermore, agricultural workers have been shown to be at higher risk for depression (Reed and Claunch, 2020), presenteeism (de Siqueira et al., 2023), obesity (Hunsucker and Reed, 2021), chronic obstructive pulmonary disease (Fontana et al., 2017), and suicide (Reed and Claunch, 2020). Moreover, female agricultural workers have an increased risk of developing breast cancer (Mills et al., 2019). These mental and physical risks may contribute to agricultural workers' shorter healthy life expectancy and increased mortality risk.

In contrast, reports have shown that agricultural work is associated with positive health outcomes, such as a reduced risk of diabetes (Thelin and Holmberg, 2014), reduced risk of frailty and death (Abe et al., 2020), and maintained overall health (Iijima et al., 2018). In addition, a verified report on Japanese adults aged 65 and older found that older adults who had worked in agriculture required less support and long-term care than those without such work experience (Haruyama et al., 2020).

However, many of the studies reported to date are limited by their cross-sectional design (Beseler and Rautiainen, 2023; Fontana et al., 2017; Hunsucker and Reed, 2021; Iijima et al., 2018; Mills et al., 2019), exploration of a single region (Abe et al., 2020; Haruyama et al., 2020; Iijima et al., 2018), or lack of focus on older adults (Fontana et al., 2017; Reed and Claunch, 2020; de Siqueira et al., 2023; Thelin and Holmberg, 2014). Even when older adults are included, such studies often fail to adequately consider differences between early and late older adulthood or gender differences (Abe et al., 2020; Haruyama et al., 2020; Iijima et al., 2018). As a result, few large-scale longitudinal studies specifically examine the impact of agricultural work on the health of older adults.

This study aims to address this research gap by analyzing six-year longitudinal data from Japan to clarify associations between various healthy ageing outcomes—including dementia, functional disability, healthy life expectancy, and mortality—and four employment categories (agricultural workers, non-agricultural workers, retired, and never worked), while considering the influences of sex, age, and urbanicity.

## 2 Material and methods

### 2.1 Data

This study uses longitudinal data collected from 2013 to 2019 as part of the Japan Gerontological Evaluation Study (JAGES; Kondo, 2016; Kondo and Rosenberg, 2018). JAGES is one of the few large-scale longitudinal studies focusing on the social determinants of health in older adults. It uses a self-administered questionnaire for those aged 65 and older who are physically and cognitively independent.

**Fig. 1:**
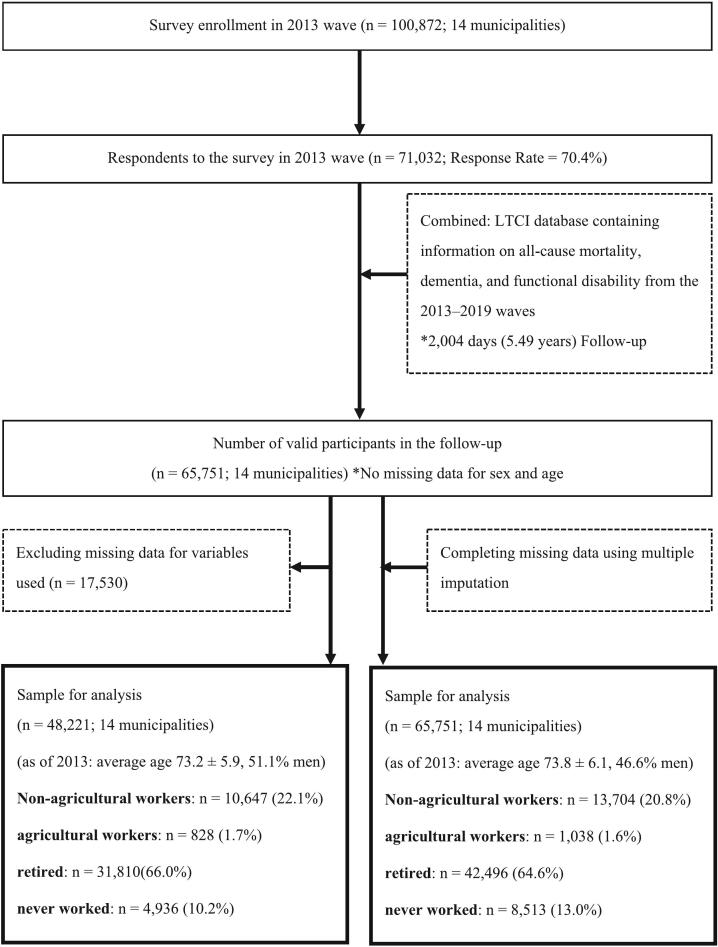
Flowchart: Flowchart of participant selection and inclusion criteria for Japanese adults aged 65 and older using the Japan Gerontological Evaluation Study, 2013–2019. Population Density Classification: Metropolitan: Areas with a population density of 4000 or more people per square kilometer. Urban/Suburban: Areas with a population density of 1000–3999 people per square kilometer. Rural: Areas with a population density of 999 or fewer people per square kilometer.

Fig. 1 shows a sample flowchart. The data include 65,751 older adults from 14 municipalities who responded to a 2013 questionnaire, linked with a national long-term care insurance (LTCI) database on mortality, dementia, and functional disability (2013–2019). After excluding 17,530 questionnaire responses due to missing explanatory variables such as activities of daily living, marital status, family structure, household income, education history, and urbanicity, we identified 48,221 valid responses for analysis. Respondents were informed that participation in the study was voluntary and that completing and returning the questionnaire indicated consent.

We conducted this study in accordance with the principles of the Declaration of Helsinki. We obtained approval from the ethics committee of Chiba University on December 11, 2020 (approval number: 3442), National Institute of Longevity Sciences on December 18, 2020 (approval number: 1274–2), and JAGES Organization on October 10, 2020 (approval number: 2019–01). Additionally, we followed the local government's guidelines for handling personal information.

### 2.2 Dependent variables

We defined the following five events as dependent variables, which could occur at any time during the six-year follow-up period: the onset of dementia (Ministry of Health, L. and W, 2010; Tsuji et al., 2019), functional disability (any level) (Ministry of Health, L. and W, 2024; Ukawa et al., 2020), functional disability (level 2 or higher) (Ministry of Health, L. and W, 2019; Ministry of Health, L. and W, 2024), loss of healthy life expectancy (Ministry of Health, L. and W, 2019), and death (Supplement A).

Dementia was defined based on the activities of daily living independence assessment criteria for older adults with dementia developed by the Ministry of Health, Labour and Welfare of Japan. Cognitive decline was categorized into five levels: I, II (IIa, IIb), III (IIIa, IIIb), IV, and M. Individuals classified as IIa or higher and certified as requiring Level 2 or higher care were defined as having dementia (Ministry of Health, L. and W, 2010) (Supplement A). Functional disability was defined as a condition requiring 25–32 min of daily care for at least one year as functional disability (any level), and a more severe condition requiring 50–70 min or more of daily care as functional disability (level 2 or higher) (Ministry of Health, L. and W, 2010.; Ukawa et al., 2020) (Supplement A). Healthy life expectancy was defined as the period until the certification of functional disability (level 2 or higher) or death (Ministry of Health, L. and W, 2019, Ministry of Health, L. and W, 2024.) (Supplement A).

The conditions are based on nationwide standards for certification of assistance and long-term care needs. A visiting surveyor determines these conditions, which are then confirmed by a written opinion from an attending physician (Tsutsui and Muramatsu, 2005). The assessment of dementia and functional disability follows a standardized, multistep assessment protocol (Kanamori et al., 2014; Ministry of Health, L. and W, 2010, Ministry of Health, L. and W, 2024.; Otsuka et al., 2018). We obtained data on dementia, functional disability, death, transfers to other municipalities, and more from the LTCI database of each municipality. Many epidemiological researchers in Japan use these criteria to determine the onset of cognitive and physical disability (Kanamori et al., 2014; Ministry of Health, L. and W, 2010; Otsuka et al., 2018; Tsuji et al., 2019; Tsutsui and Muramatsu, 2005; Ukawa et al., 2020).

### 2.3 Independent variables

We defined the following four employment statuses of older adults as the independent variables: non-agricultural workers, agricultural workers, retired, and never worked. These categories were based on their responses about their work status in 2013 and the type of work they had performed the longest.

To determine whether a respondent was working in 2013, we asked, “Which of the following describes your current work status?” The residents could select (1) working, (2) retired and not currently working, or (3) never had a job. We then asked, “In what field of work did you work the longest?” with options being (1) professional/technical, (2) managerial, (3) clerical, (4) sales/service, (5) technical/laborer, (6) agriculture, forestry, or fishing, (7) self-employed in an occupation other than agriculture, forestry, or fishing, (8) other, or (9) never had a job.

Based on their responses, we classified the respondents into three categories. Those employed in agriculture, forestry, or fishing were classified as agricultural workers (Kanamori et al., 2021). Those employed in other sectors, such as professional/technical, managerial, clerical, and sales/service, and self-employed (excluding agriculture, forestry, or fishing), were classified as non-agricultural workers. Finally, those who had never had a job were classified as never worked. Respondents who indicated that they were retired and not currently employed were classified as retired (Supplement B).

### 2.4 Covariates

We adjusted for the following factors, which are considered significant confounders in the relationship between labor, agriculture, and healthy ageing (Abe et al., 2020; Grzywacz et al., 2010; Haruyama et al., 2020). The covariates used in this study include (1) sex, (2) age, (3) activities of daily living (independent), (4) marital status, (5) family structure (living alone), (6) household income (≥2 million yen/year) in 2013, (7) education history (≥10 years), and (8) population density per square kilometer (metropolitan, urban/suburban, and rural; Abe et al., 2020; Grzywacz et al., 2010; Haruyama et al., 2020; Noguchi et al., 2022). We treated age as a binary variable, with “under 75 years old (<75)” and “75 years old and above (≥75).” Additionally, we calculated population density at the municipality level by dividing the population of each of the 14 municipalities by the habitable land area. We classified population density as follows: areas with 4000 people or more per square kilometer were metropolitan, those with 1000–3999 people per square kilometer were urban/suburban, and those with 999 people or less per square kilometer were rural (Noguchi et al., 2022).

### 2.5 Statistical analysis

We calculated baseline characteristics stratified by employment status at baseline for *n* = 48,221, presenting categorical variables as n (%). We applied regression models while adjusting for confounders to examine the association between non-agricultural workers, agricultural workers, and never worked with each outcome compared to retired. We used logistic regression for outcomes with less than 10 % prevalence and modified Poisson regression for outcomes with 10 % or greater prevalence to calculate odds ratios (OR) and risk ratios (RR). Furthermore, we confirmed 95 % confidence intervals (“CI”) (VanderWeele et al., 2020; Zou, 2004).

Because the self-administered questionnaire contained missing data (ranging from 2.2 % for education history to 16.2 % for household income), we used multiple imputations. We analyzed each of the 20 datasets using multiple imputation and combined the effect estimates using Rubin's rule (Carpenter, 2004; Rubin, 2004).

We performed four additional analyses. First, we analyzed the interaction between employment status and sex and conducted sex-stratified analyses to account for sex differences. Second, we examined the interaction between employment status and age, conducting age-stratified analyses. Finally, we divided respondents into urban (metropolitan and urban/suburban areas) and rural groups, considering the differences in agricultural practices (Ministry of Agriculture, F. and F, 2024.) and LTCI service utilization (Jin et al., 2020; Li et al., 2013). We analyzed the interaction between employment status and urban/rural areas and conducted stratified analyses based on these groups. We performed all analyses using Stata18 (StataCorp, College Station, TX, USA).

## 3 Results

The sample size for the analysis was 48,221 (male: *n* = 24,632 [51.1 %]; mean age: 73.2 ± 5.9 years). Table 1 presents descriptive statistics showing baseline characteristics stratified by employment status for 48,221 older adults in 2013. These statistics include the number and percentage of older adults for each independent variable. Of the 2013 respondents, 10,647 (22.1 %) were non-agricultural workers, 828 (1.7 %) were agricultural workers, 31,810 (66.0 %) were retired, and 4936 (10.2 %) had never worked. The incidence rates for each outcome during the 6-year follow-up period were 2.6 % for dementia, 17.3 % for functional disability (any level), 9.1 % for functional disability (level 2 or higher), 14.7 % for loss of healthy life expectancy, and 14.8 % for death.

**Table 1:** Characteristics of Japanese adults aged 65 and older stratified by work status in the 2013 wave (Before multiple imputation; *n* = 48,221).

	Work status in 2013 wave				
Characteristics	Total	Non-agricultural workers	Agricultural workers	Retired	Never worked
	48,221	10,647 (22.1)	828 (1.7)	31,810 (66.0)	4,936 (10.2)
	n (%)	n (%)	n (%)	n (%)	n (%)
Age (<75)	30,491 (63.2)	8913 (83.7)	526 (63.5)	18,806 (59.1)	2246 (45.5)
Sex (Male)	24,632 (51.1)	6667 (62.6)	522 (63.0)	16,614 (52.2)	829 (16.8)
Activities of daily living (independent)	47,135 (97.8)	10,558 (99.2)	820 (99.0)	31,042 (97.6)	4715 (95.5)
Marital status (Married)	36,045 (74.8)	8524 (80.1)	697 (84.2)	23,833 (74.9)	2991 (60.6)
Family structure (Living alone)	41,833 (86.8)	9397 (88.3)	787 (95.1)	27,598 (86.8)	4051 (82.1)
Household income (≥2 million yen/year)	24,184 (50.2)	6249 (58.7)	460 (55.6)	15,402 (48.4)	2073 (40.2)
Education history (≥10 years)	30,846 (64.0)	7267 (68.3)	427 (51.6)	20,390 (61.0)	2762 (56.0)

Population density (per square kilometer)					
Metropolitan areas(≥4000 people/1 km^2^)	12,919 (26.8)	3165 (27.9)	19 (2.3)	8403 (26.4)	1332 (27.0)
Urban/suburban areas(1000–3999 people/1 km^2^)	23,981 (49.7)	5299 (49.8)	396 (47.8)	15,974 (50.2)	2312 (46.8)
Rural areas (≤999 people/1 km^2^)	11,321 (23.5)	2183 (20.5)	413 (49.9)	7433 (23.4)	1292 (26.2)

Dementia	1274 (2.6)	110 (1.0)	15 (1.8)	944 (3.0)	205 (4.2)
Functional disability (Any level)	8324 (17.3)	841 (7.9)	93 (11.2)	6057 (19.0)	1333 (27.0)
Functional disability (Level 2 or higher)	4399 (9.1)	428 (4.0)	63 (7.6)	3234 (10.2)	674 (13.7)
Loss of healthy life expectancy	7136 (14.8)	806 (7.6)	100 (12.1)	5261 (16.5)	969 (19.6)
Death	4399 (9.1)	540 (5.1)	67 (8.1)	3263 (10.3)	529 (10.7)

Population Density Classification:

Metropolitan: Areas with a population density of 4000 or more people per square kilometer.

Urban/Suburban: Areas with a population density of 1000–3999 people per square kilometer.

Rural: Areas with a population density of 999 or fewer people per square kilometer.

Table 2 shows the multivariate analysis after multiple imputation (*n* = 65,751) showing the association with each outcome (dementia, functional disability, healthy life years lost, and death) and working status compared with retirees. Compared with retirees, non-agricultural and agricultural workers had significantly lower rates of dementia, functional disability, loss of healthy life expectancy, and death. For agricultural workers, the OR and RR for outcomes were: dementia (OR: 0.45, 95 % CI: 0.30–0.67); functional disability (any level; RR: 0.64, 95 % CI: 0.54–0.76); functional disability (level 2 or higher; RR: 0.65, 95 % CI: 0.49–0.86); loss of healthy life expectancy (RR: 0.69, 95 % CI: 0.58–0.84); and death (OR: 0.68, 95 % CI: 0.55—0.83).

**Table 2:** Association between work status and healthy ageing in Japanese adults aged 65 and older over 6 years (2013–2019) (After multiple imputation; sample size n = 65,751).

Dependent variables	OR/RR	95 %CI
Dementia		
Ref. (retired)	1.00	
Non-agricultural workers	0.63	(0.52–0.77)
Agricultural workers	0.45	(0.30–0.67)
Never worked	1.17	(1.01–1.34)

Functional disability (Any level)		
Ref. (retired)	1.00	
Non-agricultural workers	0.66	(0.63–0.70)
Agricultural workers	0.64	(0.54–0.76)
Never worked	1.08	(1.03–1.13)

Functional disability (Level 2 or higher)		
Ref. (retired)	1.00	
Non-agricultural workers	0.60	(0.55–0.66)
Agricultural workers	0.65	(0.49–0.86)
Never worked	1.12	(1.01–1.23)

Loss of healthy life expectancy		
Ref. (retired)	1.00	
Non-agricultural workers	0.66	(0.62–0.71)
Agricultural workers	0.69	(0.58–0.84)
Never worked	1.10	(1.03–1.17)

Death		
Ref. (retired)	1.00	
Non-agricultural workers	0.64	(0.57–0.71)
Agricultural workers	0.68	(0.55–0.83)
Never worked	1.15	(1.05–1.26)

Abbreviations: Ref, Reference.

Dependent Variables: The following five events occurring during the approximately 6-year follow-up period: onset of dementia, functional disability (any level), functional disability (Level 2 or higher), loss of healthy life expectancy, and death.

Dementia: Individuals classified as Rank IIa or higher and certified as requiring Level 2 or higher care.

Functional disability (any level): A condition requiring 25–32 min of daily care for at least one year.

Functional disability (Level 2 or higher): A more severe condition requiring 50–70 min or more of daily care.

Healthy life expectancy: The period until the certification of “functional disability (level 2 or higher)” or death.

Independent Variables: working (non-agricultural workers, agricultural workers), retired, and never worked in 2013 wave. The reference value has been set to be retired.

Covariates: (1) sex and (2) age, (3) activities of daily living (ADL), (4) marital status, (5) family structure (living alone), (6) household income (≥2 million yen/year) in 2013, (7) education history (≥10 years), and (8) Population density; per square kilometer (metropolitan areas, urban/suburban areas, rural areas) in 2013 wave.

Independent Variables and covariates were entered simultaneously.

As the self-administered mailed questionnaires contained missing data, multiple imputation was used to analyze the data. The multiple imputation method analyzed each of the 20 datasets and combined the effect estimates using Rubin's rule.

Population Density Classification:

Metropolitan: Areas with a population density of 4000 or more people per square kilometer.

Urban/Suburban: Areas with a population density of 1000–3999 people per square kilometer.

Rural: Areas with a population density of 999 or fewer people per square kilometer.

The first additional analyses results indicate that the interaction between employment type and sex was significant for death among non-agricultural workers and for functional disability (any level) for agricultural workers. When stratified by sex, male and female non-agricultural workers exhibited significantly lower levels of mortality, with a particularly notable reduction among men. Male and female agricultural workers had a lower risk of functional disability, with a particularly notable reduction among men (Supplement C).

The second additional analysis demonstrated that among non-agricultural workers, the interaction between employment type and age was significant for functional disability (any level), functional disability (level 2 or higher), loss of healthy life expectancy, and mortality. When stratified by age, these four outcomes were significantly lower for those under 75 years of age and those 75 and older, with a particularly notable reduction for those under 75 (Supplement D). For agricultural workers, no interaction was identified, and the stratified analysis yielded results that were largely consistent with the main findings (Supplement E).

For the third additional analysis, no interaction between employment status and urbanization was observed for non-agricultural or agricultural workers. The stratified analysis by degree of urbanization produced results that were nearly identical to the main findings (metropolitan areas and urban/suburban areas or rural areas; Supplement E).

## 4 Discussion

Using longitudinal data from the 65,751 older adults in 14 municipalities, we examined the association between the four categories of older adults (non-agricultural worker, agricultural worker, retired, and never worked) with the five outcomes (dementia, functional disability [any level], functional disability [level 2 or higher], loss of healthy life expectancy, and death). After adjusting for the eight variables, we found that non-agricultural workers and agricultural workers had a lower risk of developing dementia, functional disability (any level), functional disability (level 2 or higher), loss of healthy life expectancy, and death within three years than retired. These findings suggest that non-agricultural and agricultural workers have better health outcomes compared to retired workers.

Previous studies support our finding that work reduces the risk of dementia and functional impairment among older adults (Tomioka et al., 2018). Systematic reviews have shown that continued working in old age reduces the risk of death (Murayama et al., 2022). These findings are consistent with our study's results on the association between non-agricultural and agricultural workers and health. Furthermore, longitudinal studies have reported that physical labor, including agricultural work, lowers the risk of cognitive decline (He et al., 2021). Moreover, social participation, including agricultural labor, has reduced mortality risk (Iijima et al., 2018). These reports support our findings that agricultural workers, compared with retirees, have a lower risk of dementia and mortality. However, these studies do not clarify whether agricultural work is also associated with a lower risk of dementia, functional disability (Tomioka et al., 2018), or death (Murayama et al., 2022). Moreover, because these studies consider the association between multiple factors beyond just agricultural work and health, it is not necessarily true that agricultural work is associated with dementia (He et al., 2021; Iijima et al., 2018). Furthermore, one previous study cannot be generalized as it included only older adults in a single community (Abe et al., 2020). Therefore, this study included older adults aged 65 and older (48,221 people) with a wide range of characteristics in 14 municipalities. It is the first study to show that non-agricultural workers display a significantly lower risk for dementia, functional disability (any level), functional disability (level 2 or higher), loss of healthy life expectancy, and death than retired.

This study also provides valuable insights into how delayed retirement is associated with the healthy ageing of older adults. A systematic review shows that delaying retirement improves health outcomes and lowers mortality risk among older adults (Baxter et al., 2021). Cross-sectional studies also report that late retirement among agricultural workers positively correlates with better physical and cognitive functions (Li et al., 2021). This study investigates the association between employment and health in adults aged 65 and older who have exceeded Japan's statutory retirement age. The findings support previous studies (Baxter et al., 2021; Li et al., 2021) and suggest that farming may be one occupation that enables older adults to maintain their health while continuing to work beyond retirement age.

Several mechanisms may explain the healthy ageing benefits of employment, particularly in agriculture. Studies have reported that people living in rural areas are more physically active than those living in urban areas (Mazocco et al., 2019; Wendt et al., 2022). This suggests that agricultural workers may be more physically active. The increased physical activity of agricultural workers may explain their lower risk of dementia, functional disability (any level), functional disability (level 2 or higher), loss of healthy life expectancy, and death. Previous studies have shown that higher frequency of physical activity is associated with a lower risk of physical decline(Wong et al., 2003), depression (Mammen and Faulkner, 2013), and dementia (Sato et al., 2021), which are conditions that cause older adults to require support and care. Furthermore, frequent exercise reduces not only reduces these risks but also the risk of needing assistance and long-term care (Saito et al., 2019), which, in turn, reduces overall long-term care expenditures (Hirai et al., 2021). Additionally, older adults with higher levels of physical activity have higher self-rated health (Bize et al., 2007), and those with higher self-rated health has a lower risk of death (Manderbacka et al., 2003; Stoddard et al., 2019). These mechanisms add to previous findings that work, especially when involving physical activity such as agriculture, benefits health and may be associated with a reduced risk of dementia, functional disability (any level), functional disability (level 2 or higher), loss of healthy life expectancy, and death.

In this study, we conducted stratified analyses to examine the interactions according to sex, age, and urbanicity to verify the robustness of the main results (Supplement C, D, E). The results were generally consistent; however, the small sample size of non-agricultural workers resulted in a wide 95 % CIs for many outcomes. To more precisely analyze the influence of sex, age, and urbanicity, larger sample sizes are required.

The strength of this study lies in its use of large-scale longitudinal data from Japan to examine the impact of agricultural labor on the healthy ageing of older adults. Notably, this approach addresses the research gap identified in previous studies (Abe et al., 2020; Beseler and Rautiainen, 2023; Fontana et al., 2017; Haruyama et al., 2020; Hunsucker and Reed, 2021; Iijima et al., 2018; Mills et al., 2019; Reed and Claunch, 2020; Siqueira et al. de Siqueira et al., 2023). We demonstrated that agricultural workers aged 65 and older have a lower risk of dementia, functional disability (any level), functional disability (level 2 or higher), loss of healthy life expectancy, and death compared with retired.

This study has four limitations. First, this study defined agricultural workers as older adults who were working in 2013 and who reported that their longest-held job was in agriculture, forestry, or fishing. This definition did not allow us to distinguish between those who worked in forestry or fishing from those who worked in agriculture. Additionally, we could not exclude the possibility that some respondents were working in a job other than agriculture in 2013, although this likelihood is very low. Secondly, our study faced sampling bias because we targeted older adults who responded to a self-administered questionnaire. Kojima et al. (2022) found that individuals who do not respond to self-administered questionnaires have a higher risk of needing long-term care than those who did respond. However, this study achieved a relatively high response rate of 70.4 %, thus mitigating some concerns about this bias. Third, older adults still working as agricultural workers in 2013 may have simply been healthier individuals capable of continuing to work. As a result, it cannot be excluded that our conclusions represent either an overestimation or an underestimation of the health status of agricultural workers. Finally, we used a large dataset of 48,221 individuals with diverse characteristics from 14 municipalities across Japan. This extensive dataset makes our study more generalizable and valid compared with previous studies conducted in a single region (Abe et al., 2020; Haruyama et al., 2020; Iijima et al., 2018). However, because our study focused on the Japanese population, the findings may not be generalizable to other countries.

## 5 Conclusion

This study examined the association between employment status and healthy ageing outcomes (dementia, functional disability, loss of healthy life expectancy, death) in older adults across 14 municipalities.

Results indicate that non-agricultural and agricultural workers had significantly lower risks of dementia, functional disability, loss of healthy life expectancy, and death compared to retired workers. Labor, including agricultural work, may help maintain and improve older adults' health, providing important insights into civic participation and employment as interventions in AFCC policies. Additionally, this study shows the potential to create environments that align with the SDGs by enabling older adults to sustain employment and maintain good health and well-being.

## Funding sources

This study used data from JAGES (the Japan Gerontological Evaluation Study). This study was supported by Grant-in-Aid for Scientific Research [grant numbers 19K02200, 20H00557, 20H03954, 20K02176, 20K10540, 20K13721, 20K19534, 21H03153, 21H03196, 21K02001, 21K10323, 21K11108, 21K17302, 21K17308, 21K17322, 22H00934, 22H03299, 22J00662, 22J01409, 22K04450, 22K10564, 22K11101, 22K13558, 22K17265, 22K17409, 23K16320, 23H00449, 23H03117] from JSPS (10.13039/501100001691Japan Society for the Promotion of Science); Health Labour Sciences Research Grants [grant numbers 19FA1012, 19FA2001, 21FA1012,22FA2001, 22FA1010, 22FG2001]; the Research Funding for Longevity Sciences from 10.13039/501100007312National Center for Geriatrics and Gerontology [grant number 21–20]; 10.13039/501100009028Research Institute of Science and Technology for Society [grant number JPMJOP1831] from the Japan Science and Technology (JST); a grant from Japan Health Promotion & Fitness Foundation; contribution by Department of Active Ageing, 10.13039/100012833Niigata University Graduate School of Medical and Dental Sciences (donated by Tokamachi city, Niigata); TMDU priority research areas grant; and National Research Institute for Earth Science and Disaster Resilience. The views and opinions expressed in this article are those of the authors and do not necessarily reflect the official policy or position of the respective funding organizations.

## CRediT authorship contribution statement

**Hiroki Takeuchi:** Writing – original draft, Methodology, Formal analysis, Conceptualization. **Kazushige Ide:** Writing – review & editing, Formal analysis. **Hequn Wang:** Writing – review & editing, Methodology. **Motoki Tamura:** Writing – review & editing. **Katsunori Kondo:** Writing – review & editing, Supervision, Project administration.

## Declaration of competing interest

The authors declare that they have no known competing financial interests or personal relationships that could have appeared to influence the work reported in this paper.

## Data Availability

The data were obtained from the JAGES. All inquiries regarding these data should be directed to the Data Management Committee (e-mail: dataadmin.ml@jages.net). All JAGES data sets contain confidential information on subjects, and ethical and legal restrictions are imposed on their release. These restrictions are used to define the guidance of the local authorities who contributed to the study. Researchers may use the data by submitting a research plan (JAGES Data Use) and obtaining approval it from JAGES.
